# A Tissue-Engineered Construct Based on a Decellularized Scaffold and the Islets of Langerhans: A Streptozotocin-Induced Diabetic Model

**DOI:** 10.3390/life14111505

**Published:** 2024-11-19

**Authors:** Victor I. Sevastianov, Anna S. Ponomareva, Natalia V. Baranova, Aleksandra D. Belova, Lyudmila A. Kirsanova, Alla O. Nikolskaya, Eugenia G. Kuznetsova, Elizaveta O. Chuykova, Nikolay N. Skaletskiy, Galina N. Skaletskaya, Evgeniy A. Nemets, Yulia B. Basok, Sergey V. Gautier

**Affiliations:** 1 The Shumakov National Medical Research Center of Transplantology and Artificial Organs, 123182 Moscow, Russia; viksev@yandex.ru (V.I.S.); barnats@yandex.ru (N.V.B.); sashak1994@mail.ru (A.D.B.); allanik64@yandex.ru (A.O.N.); kuzeugenia@gmail.com (E.G.K.); nskaletsky@mail.ru (N.N.S.); skalink@mail.ru (G.N.S.); evgnemets@yandex.ru (E.A.N.); bjb2005@mail.ru (Y.B.B.); priemtranspl@yandex.ru (S.V.G.); 2 The Institute of Biomedical Research and Technology (IBRT), Autonomous Non-Profit Organization, 123557 Moscow, Russia; 3 The Sechenov First Moscow State Medical University, 119435 Moscow, Russia

**Keywords:** decellularized scaffold, streptozotocin-induced diabetes mellitus, islets of Langerhans, islet transplantation

## Abstract

Producing a tissue-engineered pancreas based on a tissue-specific scaffold from a decellularized pancreas, imitating the natural pancreatic tissue microenvironment and the islets of Langerhans, is one of the approaches to treating patients with type 1 diabetes mellitus (T1DM). The aim of this work was to investigate the ability of a fine-dispersed tissue-specific scaffold (DP scaffold) from decellularized human pancreas fragments to support the islets’ survival and insulin-producing function when injected in a streptozotocin-induced diabetic rat model. The developed decellularization protocol allows us to obtain a scaffold with a low DNA content (33 [26; 38] ng/mg of tissue, *p* < 0.05) and with the preservation of GAGs (0.92 [0.84; 1.16] µg/mg, *p* < 0.05) and fibrillar collagen (273.7 [241.2; 303.0] µg/mg, *p* < 0.05). Rat islets of Langerhans were seeded in the obtained scaffolds. The rats with stable T1DM were treated by intraperitoneal injections of rat islets alone and islets seeded on the DP scaffold. The blood glucose level was determined for 10 weeks with a histological examination of experimental animals’ pancreas. A more pronounced decrease in the recipient rats’ glycemia was detected after comparing the islets seeded on the DP scaffold with the control injection (by 71.4% and 51.2%, respectively). It has been shown that the DP scaffold facilitates a longer survival and the efficient function of pancreatic islets in vivo and can be used to engineer a pancreas.

## 1. Introduction

Type 1 diabetes mellitus (T1DM) is a chronic autoimmune disease characterized by absolute insulin deficiency due to the death of β-cells, which leads to hyperglycemia and the development of severe diabetic complications (angiopathy, neuropathy, nephropathy, etc.) [1]. A modern method of treating severe cases of type I diabetes is pancreatic islet transplantation according to the Edmonton Protocol [2,3,4,5], which requires a significant mass of islets from several donors [6]. The replacement of dead β-cells by islet transplantation makes it possible to establish stable euglycemia in patients [7], improve the quality of life, and reduce the risk of secondary complications compared to insulin therapy [8].

However, the shortage of donor organs and the islets’ limited functioning time in vivo stimulate the search for tissue engineering and regenerative medicine technologies aimed at the long-term preservation of their viability and functional activity. During the process of isolation, culturing, and transplantation, the islets lose vascularization, innervation, and connection with the extracellular matrix (ECM), which makes them even more susceptible to oxidative stress [9]. In addition, post-transplant islet injury is associated with blood-mediated immediate inflammatory reactions, hypoxia, and the toxic effects of immunosuppressants [10,11,12].

At present, the improvement of biotechnology methods allows us to hope for the prospects of using technologies based on the creation of a tissue-engineered pancreas, formed on the basis of insulin-producing cells that maintain viability and functional activity for a long time and a biocompatible scaffold that provides them with the best conditions. The advantage of using islets isolated from the donor pancreas to create a tissue-engineered pancreas over insulin-producing cells of other origins is that β-cells retain paracrine connections with all islet cell types [13].

The important components of a tissue-engineered pancreas are the ECM components of the scaffold that prevent cellular stress and promote the maintenance of islet viability and function. Scaffolds with ECM components are universal platforms that provide structural and mechanical support to the islets and also serve as a reservoir of growth factors, cytokines, and antioxidants, and transmit signals to islet cells via integrins [14,15,16].

Previous studies to obtain islet scaffolds focused on the scaffold construction based on synthetic and natural polymers, but none of them could accurately simulate the complexity of the actual pancreatic tissue composition and structure [17,18,19].

It was shown that when cultured on scaffolds containing natural ECM components, the islets retained viability and secretory activity longer versus culturing the islets alone [20,21]. The transplantation of the islets cultured on such scaffolds was more successful [22].

Recently, pancreas decellularization technologies have been used as an alternative to obtaining a scaffold with the preserved structural and compositional features of a pancreatic tissue. When developing protocols for obtaining a tissue-specific scaffold from a decellularized pancreas, it is important to take into account the preservation of native ECM components in it for its functional activity, such as structural proteins, glycoproteins, and cell adhesion factors, with the maximum removal of DNA to minimize the immune response during the implantation of a tissue-engineered pancreas [23,24,25,26,27]. A scaffold with the preserved biochemical composition and spatial structure of a native ECM can be used as a framework for the subsequent recellularization by insulin-producing cells and further implantation [28,29].

It was found that islets cultured with a tissue-specific scaffold obtained as a result of decellularizing both the whole pancreas and pancreatic tissue fragments increased insulin secretion versus isolated islets in monoculture [28,30]. Wu et al. [31] showed in an experimental T1DM model that a scaffold from the ECM pancreatic tissue recellularized with a population of insulin-producing cells was capable of controlling blood glucose levels in mice versus the same cells cultured in Petri dishes.

It should be noted that the success of in vivo studies depends on the choice of the T1DM model; therefore, despite their variety, the choice of the most adequate and accessible model is an independent issue. The most commonly used substances are streptozotocin (STZ) and alloxan [32]—toxic drugs structurally similar to the glucose molecule, which are accumulated in β-cells via the glucose transporters GLUT4 and GLUT2, respectively.

Previously, we described approaches to obtaining tissue-specific scaffolds from the decellularized rat [33] and human [30] pancreas. The preservation of structure and the prolonged viability and functionality of the islets cultured with a tissue-specific scaffold versus the islets cultured alone was demonstrated.

Thus, the functionality of an in vitro tissue-engineered pancreas is directly related to the integrity of the cellular component that it contains, which may ultimately be of critical importance in vivo [34].

The aim of this study was to investigate the ability of decellularized pancreas fragments to support the islets’ survival and insulin-producing function when injected in a streptozotocin-induced diabetic rat model.

## 2. Materials and Methods

### 2.1. Obtaining Technology of the DP Scaffold

A fine-dispersed scaffold (DP scaffold) was obtained from decellularized pancreatic tissue as a result of human pancreatic fragments’ decellularization according to a previously developed protocol [30]. Briefly, human pancreatic fragments underwent 3 cycles of freezing up to −80 °C and thawing to +37 °C, with the subsequent mechanical tissue disintegration to 2 × 1 × 1 mm. Tissue fragments were treated in three changes in phosphate-buffered saline (PBS) (pH = 7.4), containing 0.1% SDS solutions and increasing concentrations of Triton X100 (1%, 2% and 3%, respectively) (Sigma-Aldrich, St. Louis, MO, USA). In each solution, the sample was kept for a day at room temperature under the conditions of constant stirring at a speed of 1.0 rpm on the CellRoll roller system (INTEGRA Biosciences AG, Zizers, Switzerland). Then, the tissue was thoroughly rinsed for 72 h in three changes in antibiotic–antimycotic containing PBS (ampicillin, 10.0 μg/mL, and amphotericin B, 1.5 μg/mL). The obtained samples of the finely dispersed scaffold (DP scaffold) were placed in 10 mg cryotubes, frozen and γ-sterilized (1.5 Mrad).

### 2.2. Biochemical Study of Scaffold from Decellularized Human Pancreas (DP Scaffold)

#### 2.2.1. DNA Concentration Determination

To quantify the DNA content in the samples of a native human pancreas (*n* = 5, 10 mg each) and a DP scaffold (*n* = 5, 10 mg each), a DNA extraction kit DNeasy Blood&Tissue Kit (QIAGEN, Hilden, Germany) was used according to the manufacturer’s instructions. To quantify double-stranded DNA, a kit with a fluorescent dye, QuantiT PicoGreen dsDNA Assay Kits and dsDNA Reagents (Invitrogen, Carlsbad, CA, USA), was used in accordance with the manufacturer’s instructions. A Tecan Spark 10 M tablet reader (Tecan Trading AG, Männedorf, Switzerland) was used at a wavelength of 520 nm to calculate the amount of DNA in the samples.

#### 2.2.2. Glycosaminoglycan (GAG) Concentration Determination

To quantify GAG content in native and decellularized pancreatic tissue, the samples of a native human pancreas (*n* = 3, 10 mg each) and a DP scaffold (*n* = 3, 10 mg each) were lyophilized at a temperature of −80 °C (FreeZone, Labconco, Kansas City, MO, USA). After lyophilization, the samples weighing 30 mg each (*n* = 3) were lysed in papain solution (Sigma-Aldrich, St. Louis, MO, USA) at a temperature of +65 °C for 12 h. The concentration of GAGs was determined using a 1,9-dimethylmethylene blue (DMMB) dye (Sigma-Aldrich, St. Louis, MO, USA), using a Tecan Spark 10 M tablet reader (Tecan Trading AG, Männedorf, Switzerland) to measure absorption at a wavelength of 525 nm.

#### 2.2.3. Collagen Concentration Determination (Collagen Quantification)

The collagen content in the human pancreas and DP scaffold samples weighing 150 mg was determined using a Sircol Soluble COLLAGEN Assay (Biocolor, Belfast, UK) according to the manufacturer’s protocol. The samples were kept for 12 h at RT in pepsin (1 mg/mL in 0.01 M HCl, Sigma-Aldrich, St. Louis, MO, USA). The processed samples were added to 1 mL Sircol dye reagent and mechanically agitated for 30 min at RT, followed by centrifugation. Ice-cold AcidSalt Wash Reagent was gently layered on collagen pellets to remove unbound dye from the surface. Then, the wash was drained into a waste container. The remaining moisture was removed using a piece of paper filter. An alkali reagent (250 µL) was added to standards and samples. The absorbance was measured at a wavelength of 555 nm using a Spark 10 M microplate reader (TecanTrading AG, Männedorf, Switzerland).

#### 2.2.4. Determination of Cytotoxicity

The cytotoxicity of DP scaffold samples in vitro was evaluated in accordance with ISO 10993-5:2009 [35] on a NIH/3T3 mouse fibroblast culture (ATCC^®^CRL-1658™, American Type Culture Collection) by direct contact.

Fibroblasts were cultured in standard culture vials with a thickness of 25 cm^2^ (CELLSTAR Greiner Bio-One, Frickenhausen, Germany) in a complete growth medium containing DMEM (Dulbecco’s modified Eagle medium) with a high glucose content (4.5 g/L, DMEM high glucose with HEPES, PanEco, Moscow, Russia), 10% calf serum (Biosera, Cholet, France), 1% antibiotic–antimycotics Anti-Anti (Gibco, Thermo Fisher Scientific, Waltham, MA, USA) and 2 mM glutamine (PanEco, Moscow, Russia) in a CO_2_ incubator under standard conditions (+37 °C, humidified atmosphere containing (5 ± 1)% CO_2_). The cells were removed from the cultured plastic surface using the dissociating reagent TrypLE Express Enzyme (Gibco, Thermo Fisher Scientific, Waltham, MA, USA). The initial number of cells in the suspension was determined using the TC20TM Automated Cell Counter (Bio-Rad Laboratories Inc., Hercules, CA, USA). To study the cytotoxic effect, fibroblasts were seeded into 96-well flat-bottomed culture plates (CELLSTAR Greiner Bio-One, Frickenhausen, Germany) at a concentration of 1–2 × 10^4^ cells per well and incubated in a complete growth medium until the formation (80 ± 10)% of a monolayer under standard conditions. Then, the test (*n* = 8) and control samples were introduced into wells with a cellular monolayer and incubated for 24 h. Fibroblast culture was visually assessed using a Nikon Eclipse TS100 microscope (Nikon, Tokyo, Japan).

A DMEM nutrient medium with a high (4.5 g/L) glucose content (PanEco, Moscow, Russia) was used as a negative control sample to demonstrate the background cells’ reactions. As a positive control sample to demonstrate the appropriate reaction of the test system, the single-element aqueous zinc standard 10,000 mcg/mL (Sigma-Aldrich, St. Louis, MO, USA) was used.

The cells were also stained with a complex of fluorescent vital dye LIVE/DEAD Cell Viability/Cytotoxicity Kit (molecular probes by Life Technologies, Carlsbad, CA, USA) in accordance with the protocol recommended by the manufacturer. Microscopy was performed using a Nikon Eclipse Ti fluorescence microscope (Nikon, Tokyo, Japan). The viability of cells after contact with DP-scaffold samples was assessed by trypan blue staining and a TC20TM Automated Cell Counter (Bio-Rad Laboratories Inc., Hercules, CA, USA).

### 2.3. Rat Islets Isolation, Dithizone Staining

Male Wistar rats weighing 300–380 g (*n* = 30) from the laboratory animal nursery of “KrolInfo” LLC were used to streptozotocin-induced T1DM (recipient animals) and isolate the islets of Langerhans (donor animals). The acclimatization and maintenance of laboratory animals were carried out in accordance with the interstate standard GOST ISO 10993-2-2009 “Medical devices. Assessment of the biological effect of medical devices. Part 2. Requirements for the treatment of animals” [36].

The manipulations did not cause pain to the animals and were carried out in compliance with Russian legislation, namely GOST 33215-2014 (Guidelines for the accommodation and care of laboratory animals [37]. Rules for the equipment of premises and organization of procedures) and GOST 33216-2014 (Guidelines for the accommodation and care of laboratory animals. Rules for the accommodation and care of laboratory rodents and rabbits) [38]. The work was approved by the Local Ethics Committee at the Shumakov National Medical Research Center of Transplantology and Artificial Organs, Moscow, Russia (28 January 2021, Protocol No. 280121-1/1e).

The islets were isolated from the male Wistar rat pancreas. The animals were subjected to inhalation euthanasia using Isoflurane (Laboratories Kariozo, Carrer de les Borges Blanques, Spain); then, the pancreas was excised under sterile conditions, which was immediately placed in a Petri dish with a cold (+4 °C) Hanks’ solution without Ca^2+^ and Mg^2+^ ions (Thermo Fisher Scientific, Waltham, MA, USA) containing amphotericin B. All further manipulations requiring sterility were carried out in a laminar flow box providing sterile air. A total of 2 mL of NB1 collagenase solution (20 PZ U/g tissue activity) with neutral NP protease (1.5 DMC U/g tissue activity) was injected into pancreatic tissue by successive intraparenchymal injections (Serva, Heidelberg, Germany). The stretched pancreatic tissue, without cutting, was carefully divided into 10–12 approximately equal parts with micropincers, then transferred to a vial and incubated for 7–10 min in an orbital shaker incubator (Biosan, Riga, Latvia) at a temperature of +37 °C with a rotation speed of 150 rpm. The effect of collagenase was stopped by adding a cold (+4 °C) Hanks’ solution. The resulting small fragments were filtered through a strainer (Corning-Costar, Wilkes Barre, PA, USA) with a cell diameter of 100 microns, and the filtrate was collected in conical tubes and centrifuged for 1 min at a speed of 800 rpm. The supernatant was washed twice with fresh Hanks’ solution for 1–1.5 min at 1200–1300 rpm to obtain the islets’ suspension.

The islets were identified by dithizone staining (Sigma-Aldrich, St. Louis, MO, USA) immediately after isolation. To perform this, part of the suspension was mixed with a dithizone solution (1 mg/mL) in a volume ratio of 2:1 and incubated for 20–30 min at a temperature of +37 °C.

The freshly isolated islets were resuspended in a complete growth medium containing DMEM (glucose 1.0 g/L) (PanEco, Moscow, Russia), 10% embryonic veal serum (HyClone, Logan, UT, USA), Hepes (Thermo Fisher Scientific, Waltham, MA, USA), 2 mM alanyl-glutamine (PanEco, Moscow, Russia), 1% antibiotic–antimycotics Anti-Anti (Gibco, Thermo Fisher Scientific, Waltham, MA, USA), introduced into culture vials and cultured for 24 h under standard conditions at +37 °C in a CO_2_ incubator in a humidified atmosphere containing 5% CO_2_.

### 2.4. Cell Viability Assay

The viability of the islets cultured for 24 h was determined by fluorescent staining with AO/PI (PanEco, Moscow, Russia).

For staining, part of the islet suspension was placed in a Petri dish, mixed with a prepared working solution of the dye in a volume ratio of 2:1 and incubated in the dark for 15–30 min. The calculation of viable islets was carried out using a Nikon Eclipse 50i fluorescent microscope (Nikon, Tokyo, Japan) at a ×10 magnification of the lens.

### 2.5. Enzyme Immunoassay (ELISA)

To determine the functional activity of the islets after 24 h of culturing, the insulin content was measured under the influence of a traditional hormone secretion stimulator. To perform this, the growth medium was replaced with a fresh one with a low glucose content of 2.8 mmol/L. After 60 min of incubation under standard conditions, culture medium samples were taken. Then, the growth medium was removed and replaced with a fresh one with a high glucose concentration of 25 mmol/L. After 60 min of incubation under standard conditions, culture medium samples (*n* = 2) were also taken for an enzyme immunoassay (ELISA) using the Rat Insulin ELISA Kit (Thermo Fisher Scientific, Waltham, MA, USA).

### 2.6. Rat Model of Type 1 Diabetes Mellitus

To induce T1DM, the rats were given a fractional STZ injection (Biorbyt, New Delhi, India), intraperitoneally injected 15 mg/kg/day for 5 consecutive days (a total dose of 75 mg/kg). The drug for injection was prepared ex tempore by diluting STZ in 0.9% sodium chloride solution and injected into the peritoneal cavity. The dynamics of the glycemic level were determined daily using an Accu-Chek Active glucometer (Roche, Basel, Switzerland).

To exclude a spontaneous diabetic status reversion, the animals were monitored for the next 14 days after the last STZ injection. Fasting blood glycemia levels and body weight were monitored weekly, and the appearance and amount of water consumed by the animals were evaluated daily.

T1DM was considered stable if, 2 weeks after the last STZ dose, the blood glycemia level in rats exceeded 20.0 mmol/L.

In the rats with a glucose concentration of less than 20.0 mmol/L, the clinical signs of diabetes were poorly expressed. Such animals were not used in the experiment. The animals with a glycemic level exceeding the maximum permissible value of the glucose meter (33.3 mmol/L) were considered unsuitable for a subsequent experiment.

### 2.7. Preparation of Islets and Scaffold for Injection into Rats with Streptozotocin-Induced Diabetic Model

A concentrated islets’ suspension cultured for 24 h was obtained by centrifugation in a complete growth medium for 2 min at a speed of 1200 rpm, then rinsed from the growth medium in Hanks’ solution under the same regime. For each injection to the rats from the experimental groups, 2000 islets were selected from 1 to 2 rat pancreases and resuspended in 1.0–1.2 mL of Hanks’ solution. For each rat from experimental group 2, the selected islets were seeded in a volume of a finely dispersed sterile DP scaffold (10.0 ± 0.1 mg in 100 µL of Hanks’ solution).

An islets’ suspension (alone) and islets seeded on the DP scaffold were placed in 2 mL syringes and a 23G needle for a subsequent injection.

### 2.8. Intraperitoneal Injection of Islets of Langerhans Alone and Seeded on DP Scaffold into Rats with Streptozotocin-Induced T1DM

The study of the DP-scaffold effect on the morphofunctional state of the islets of Langerhans in vivo was carried out on rats with pronounced stable T1DM induced by daily fractional injections of STZ in small doses. The islets of Langerhans alone or seeded on a DP scaffold were injected intraperitoneally into the lower third of the abdomen.

The animals selected for the experiment were divided into groups:

1. The control group (animals without treatment)—5 rats;
2. Experimental group 1 (an intraperitoneal injection of 2000 allogeneic islets of Langerhans alone)—5 rats;
3. Experimental group 2 (an intraperitoneal injection of 2000 allogeneic islets of Langerhans seeded on a DP scaffold)—5 rats.

All animals were monitored for 10 weeks. The glycemia level in the capillary blood of animals was measured on an empty stomach weekly 12 h after the last meal.

### 2.9. Histological Staining

The identification of structural disorders in the pancreas parenchyma was carried out via a morphological examination. The extracted organs of experimental animals were fixed in 10% buffered formalin for 24 h, then dehydrated in alcohols of ascending concentrations, kept in a mixture of ethanol and chloroform, or pure chloroform, after which they were poured into paraffin.

Using the RM2245 microtome (Leica, Wetzlar, Germany), sections with a thickness of 5 microns were obtained, which were stained with hematoxylin and eosin, and with Masson’s stain for total collagen. The cellular composition of pancreatic islets in the rat pancreas from the control and experimental groups was evaluated using immunohistochemical staining of the islet cells’ main types using antibodies to insulin and glucagon (Abcam, Cambridge, UK) and the Rabbit Specific HRP/DAB (ABC) Detection IHC kit (Abcam, Cambridge, UK) imaging system.

### 2.10. Statistical Analysis

Data were analyzed with SPSS 26.0 statistical software. The distribution of variables was tested with the Shapiro–Wilk procedure. The results were compared using the Mann–Whitney unpaired *t*-test and the Kruskal–Wallis test, where *p* < 0.05 was considered statistically significant.

## 3. Results

**Figure 1:**
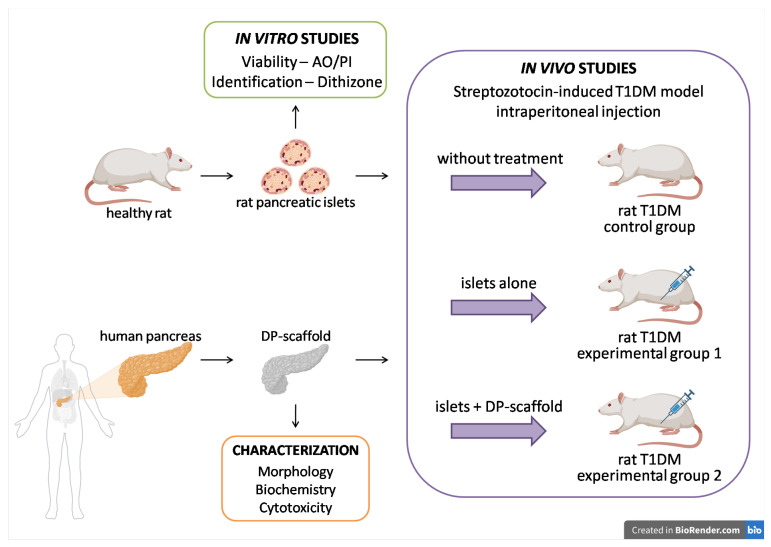
Research design.

The research design is presented in Figure 1.

### 3.1. DP-Scaffold Characterization

**Figure 2:**
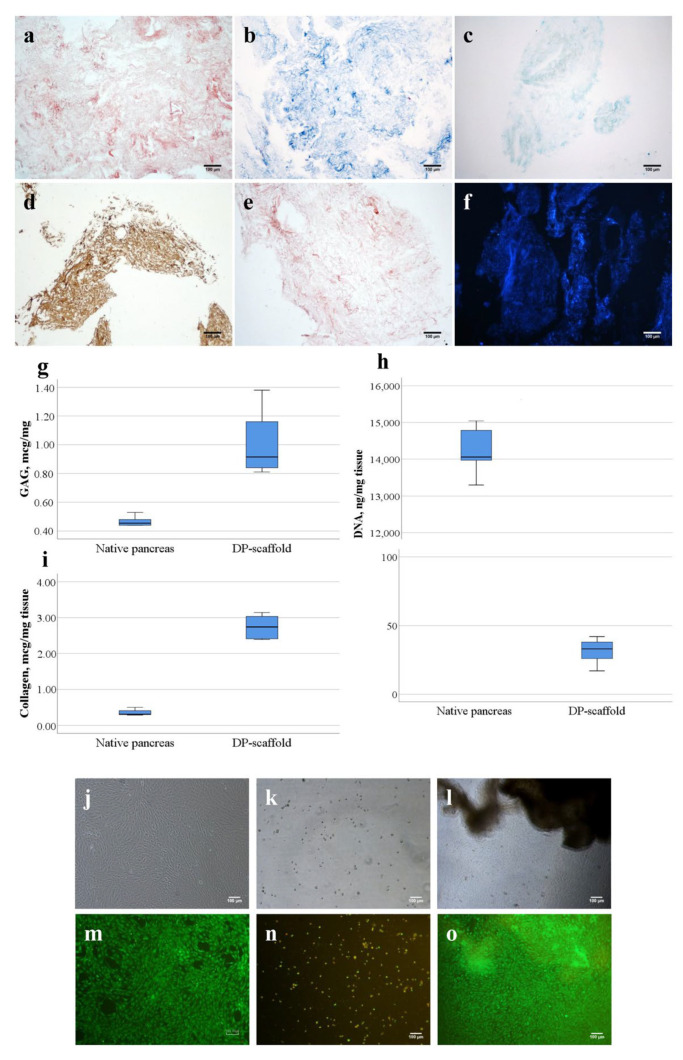
The DP-scaffold characterization. (**a**–**f**) A histological picture of a tissue-specific scaffold from a decellularized pancreas (DP scaffold); (**a**) H&E staining; (**b**) Masson’s staining; (**c**) alcian blue staining; (**d**) immunohistochemical staining with antibodies to type I collagen; (**e**) staining with orcein for elastic fibers; (**f**) DAPI staining (scale bar = 100 µm); (**g**) GAG content; (**h**) collagen content; (**i**) DNA content; (**j**–**o**) a DP-scaffold cytotoxicity study in vitro; (**j**,**m**) negative control; (**k**,**n**) positive control; (**l**,**o**) a DP scaffold on the surface of the NIH/3T3 monolayer; (**j**–**l**) phase contrast microscopy; (**m**–**o**) fluorescence LIVE/DEAD; scale bar = 100 µm.

A tissue-specific fine-dispersed scaffold was obtained as a result of a decellularization of human pancreatic fragments (DP scaffold) according to a previously developed protocol [30]. Research has shown that a DP scaffold preserves the morphofunctional properties of a native pancreatic tissue ECM, contains basic fibrillar proteins (type I collagen, elastin), glycosaminoglycans (GAGs) and has low immunogenicity (no more than 0.1% DNA) (Figure 2).

The development of a technology for the obtainment and research of a tissue-specific scaffold was approved by the Local Ethics Committee at the Shumakov National Medical Research Center of Transplantology and Artificial Organs, Moscow, Russia (16 March 2018, Protocol No. 160318-1/1e).

A quantitative analysis has shown that the DP-scaffold samples were substantially (*p* < 0.05) cleared of DNA: the DNA content decreased from 14,058 [13,970; 14,786] ng/mg of tissue to 33 [26; 38] ng/mg of tissue (Figure 2h), which is less than 50 ng/mg of tissue and indicates effective decellularization [39]. At the same time, the preservation of GAGs and fibrillar collagen in decellularized tissue was determined: 0.92 [0.84; 1.16] mcg/mg and 273.7 [241.2; 303.0] mcg/m, correspondingly (Figure 2g,i).

In vitro studies have shown that the DP-scaffold samples do not have a cytotoxic effect on the adhesion and proliferation of NIH/3T3 mouse fibroblast culture (Figure 2j–o). A microscopic examination demonstrated the absence of changes in the cellular morphology of fibroblasts after contact with the DP-scaffold samples (Figure 2l). The LIVE/DEAD staining of NIH/3T3 fibroblasts confirmed the absence of any cytotoxic effect of the DP-scaffold samples, where the green fluorescence of viable cells forming a monolayer was observed (Figure 2o). The cell viability after contact with DP-scaffold samples was 97.3% ± 1.2%, while it was 97.7% ± 1.3% in the negative control and 18.2% ± 0.4% in the positive control.

### 3.2. Rat Islets Isolated, Characterizied, and Seeded on DP Scaffold

**Figure 3:**
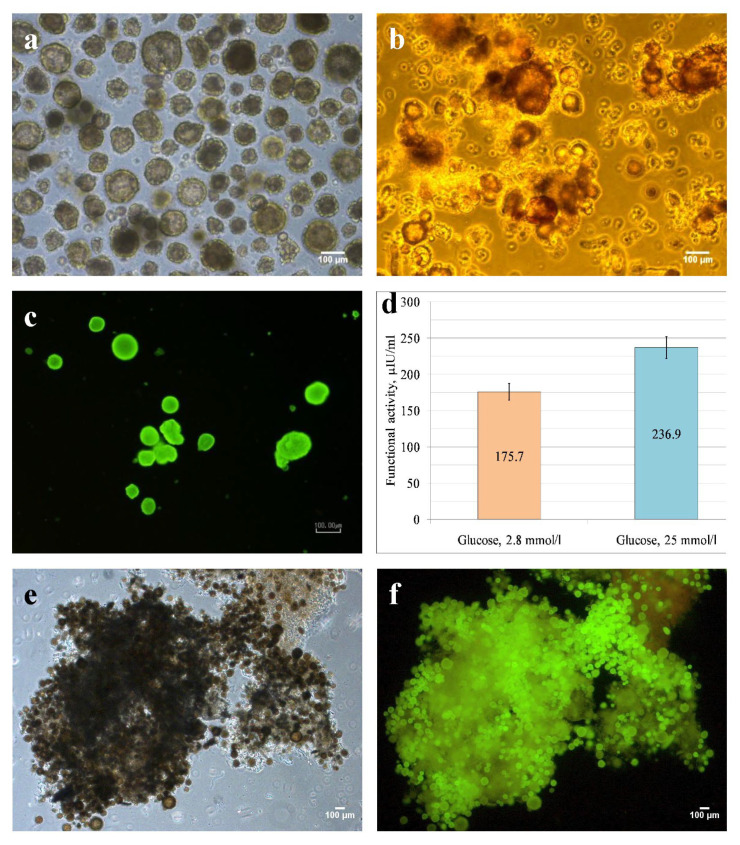
(**a**–**c**) Isolated healthy rat islets of Langerhans: (**a**) phase contrast microscopy; (**b**) dithizone staining; (**c**) islets cultured for 24 h, acridine orange and propidium iodide (AO/PI) fluorescent staining; (**d**) functional activity of isolated healthy rat islets of Langerhans cultured for 24 h; (**e**,**f**) islets seeded on DP scaffold: (**e**) phase contrast microscopy; (**f**) AO/PI fluorescent staining; scale bar = 100 µm.

Rat islets of Langerhans were isolated from the pancreas of mature male Wistar rats weighing 200 ± 20 g. The freshly isolated islets of Langerhans had a predominantly rounded shape, basically preserving the integrity of the macrostructure remaining intact during the islets’ isolation (Figure 3a), and only in some islets the signs of fragmentation and destruction were observed. The islets identified by dithizone staining selectively acquired a red-orange color, while acinar cells remained unstained (Figure 3b).

The viability of rat islets, observed after 24 h of culturing by staining with acridine orange and propidium iodide (AO/PI), was 97% (Figure 3c).

In culture medium samples taken after 24 h of islet culturing, the basal insulin concentration was 175.7 ± 11.8 µIU/mL; after stimulation with a “hyperglycemic” glucose level (25 mmol/L), it increased by 34.8% (236.9 ± 15.2 µIU/mL), which confirmed the functional activity of isolated islets (Figure 3d). The relative nature of the quantitative assessment of the cultured islets’ functional activity must be noted, since in model experiments in vitro it is impossible to reproduce conditions for insulin-producing cells similar to a native pancreas [40].

Viable functionally active islets of Langerhans exhibited adhesive properties and were randomly deposited on the scaffold surface when in direct contact with the DP scaffold (Figure 3e). Fluorescent staining with AO/PI confirmed the viability of most islets within the formed tissue construct (Figure 3f).

To achieve reliable results of the study of the DP-scaffold effect on the islets’ viability and function in vivo in a streptozotocin-induced diabetic rat model, allogeneic rat islets of Langerhans were used, which, from their morphofunctional state (the level of viability and functional activity), are suitable for injection to recipient rats with T1DM.

### 3.3. Streptozotocin-Induced Diabetic Rat Model

The T1DM model was induced by a fractional intraperitoneal injection of STZ, which allowed us to ensure a stable diabetic status in animals while excluding their death and spontaneous reversion of diabetes, not caused by the direct action of the studied antidiabetic treatment. This approach to the T1DM induction eliminates the possibility of obtaining erroneous experiment results.

**Figure 4:**
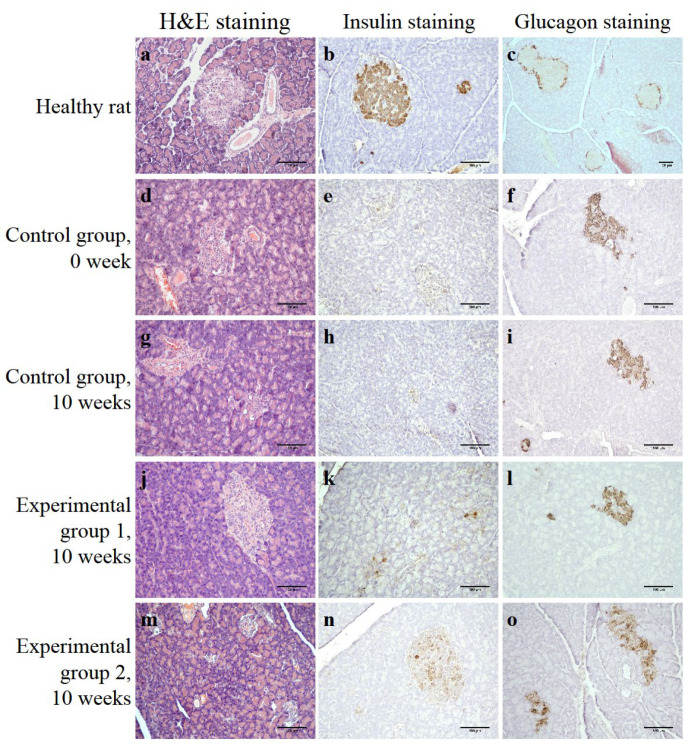
(**a**–**c**) A pancreas of a healthy rat; (**d**–**f**) a pancreas of a rat from the control group with streptozotocin-induced T1DM, 0 week; (**g**–**i**) a pancreas of a rat from the control group with streptozotocin-induced T1DM, 10 weeks; (**j**–**l**) a pancreas of a rat from experimental group 1 with streptozotocin-induced T1DM after an intraperitoneal injection of the islets of Langerhans; (**m**–**o**) a pancreas of a rat from experimental group 2 with streptozotocin-induced T1DM after an intraperitoneal injection of the islets of Langerhans with a scaffold; (**a**,**d**,**g**,**j**,**m**) H&E staining; (**b**,**e**,**h**,**k**,**n**) insulin immunohistochemical staining; (**c**,**f**,**i**,**l**,**o**) glucagon immunohistochemical staining; scale bar = 100 µm.

In the experiment, rats (*n* = 15) with stable hyperglycemic indicators in fasting blood from 20.4 mmol/L to 32.6 mmol/L were used 2 weeks after the completion of STZ injections (Figure 4a–c). Clinically, these animals showed physical inactivity, a tendency to develop wounds and ulcers, polyuria, significant polydipsia, and a decrease in body weight compared to intact animals. The rats selected for the experiment were divided into three groups: one control (*n* = 5) and two experimental groups (*n* = 10).

To confirm the T1DM development, a histological examination of the rat pancreas with a glycemic level of 24.8 mmol/L was performed 2 weeks after the last STZ injection. In general, the preservation of the exocrine parenchyma was noted; moderate focal lymphoid cell infiltration was detected only in some lobules. Vacuolized cells (probably β-cells) were detected in a few irregular-shaped islets of Langerhans (Figure 4d). When stained with antibodies to insulin, a negative reaction was observed, which indicated the β-cells’ death (Figure 4e). At the same time, glucagon-positive α-cells were found not only on the periphery, but also in the central part of the islets, and in significantly greater numbers than in a healthy rat (Figure 4f).

### 3.4. Injection Islets and Islets Seeded on DP Scaffold in STZ-Induced Diabetic Rats

The functional efficiency of islets alone and islets seeded on a tissue-specific scaffold from a decellularized human pancreas was evaluated with regard to the ability to reduce the elevated blood glucose level of rats with STZ-induced T1DM for 10 weeks.

#### 3.4.1. Control Group

**Figure 5:**
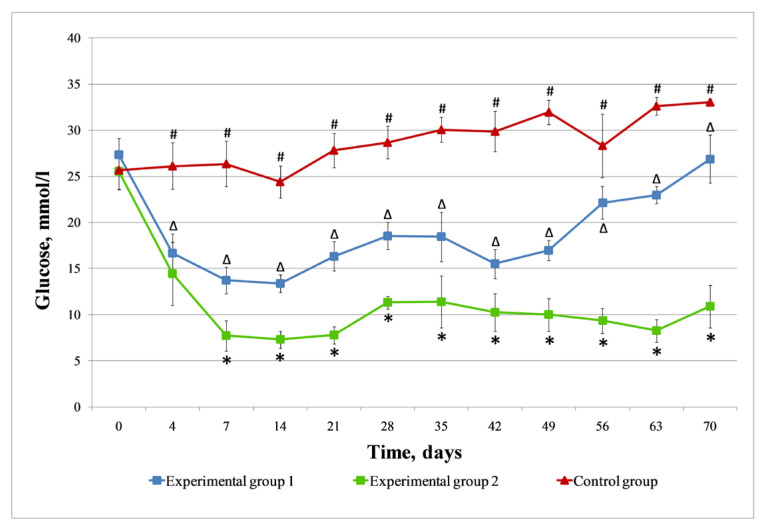
The dynamics of blood glucose levels in rats with streptozotocin-induced T1DM from the control (without treatment) and experimental groups after an intraperitoneal injection of the islets of Langerhans or the islets of Langerhans seeded on a DP scaffold. ^∆^ *p* < 0.05: control group vs. experimental group 1; ^#^ *p* < 0.05: control group vs. experimental group 2; * *p* < 0.05: experimental group 1 vs. experimental group 2.

All animals in the control group retained the pronounced clinical signs of T1DM during the observation period, and the general condition of the rats worsened. Polydipsia, polyuria, inactivity, non-healing purulent wounds on tails, and the further loss of body weight (from 285 ± 25 g to 175 ± 30 g) were noted in animals. The hyperglycemia level in the blood of the control group animals (*n* = 5) increased during the entire follow-up period (Figure 5). Of the five control rats, two died at weeks 3 and 8 of the experiment.

In histological samples of the control group rats’ pancreas, after their withdrawal from the experiment (10 weeks), a few irregular islets of Langerhans were found, with an almost complete absence of insulin-positive β-cells and numerous glucagon-positive α-cells (Figure 4g–i).

#### 3.4.2. Experimental Group 1 (Injection of Islets of Langerhans)

In experimental group 1, after an intraperitoneal injection of the islets of Langerhans alone, the T1DM characteristic clinical signs in rats (*n* = 5) slowed down after a week, and weight gain was noted (from 285 ± 25 g to 310 ± 10 g). At the same time, a significant decrease in the hyperglycemia level from 27.4 ± 1.8 mmol/L to 16.7 ± 2.1 mmol/L was noted in all recipient rats three days after the islets’ injection (Figure 5). Subsequently, the glucose content in the rats’ blood has been established at the level of 13.4 ± 1.0 mmol/L, on average 2.1 times less than the level before the islets’ injection (27.4 ± 1.8 mmol/L). However, after 7 weeks of follow-up, the glucose level in experimental group 1 began to increase and by week 10 reached a level (26.9 ± 2.6 mmol/L) close to the initial values. At week 6, a rat with a blood glucose level of 27.3 mmol/L died, despite the fact that after the islets’ injection, the rat had a stable decrease in hyperglycemia.

The histological picture of experimental group 1 rats’ pancreases revealed the good preservation of the exocrine parenchyma without signs of inflammation and irregular shape islets, as in the control group rats’ pancreases, with rare vacuolized cells (Figure 4j). During the immunohistochemical staining of insulin-positive cells, the presence of single β-cells (Figure 4k) was noted, while numerous glucagon-positive α-cells were present in the islets (Figure 4l).

#### 3.4.3. Experimental Group 2 (Injection of Islets of Langerhans Seeded on DP Scaffold)

In experimental group 2, a week after an intraperitoneal injection of the islets of Langerhans seeded on a DP scaffold, a slowdown in the clinical manifestations of T1DM was observed in rats (*n* = 5), the same as in the experimental group 1 animals. There was a prolonged decrease in the glycemia level by almost 3.5 times versus the initial value, and the achievement of normoglycemic parameters (up to 7.0 mmol/L) in individual measurements (Figure 5). By the end of the experiment (10 weeks of follow-up), blood glucose levels of 9.5, 9.1 and 7.9 mmol/L were recorded in three rats, respectively, which turned out to be much lower than the initial average level (25.5 ± 1.9 mmol/L). In the fourth week of observation, a rat from experimental group 2 with a glucose level of 5.9 mmol/L died.

A histological examination of experimental group 2 rats’ pancreases revealed the preservation of the exocrine parenchyma without inflammation signs and the absence of infiltrate (Figure 4m). A few vacuolized cells were observed in the islets, both of a rounded and irregular shape. Immunohistochemical staining for insulin revealed an increase in the number of insulin-positive β-cells in experimental group 2 rats compared to the animals from experimental group 1 (Figure 4n), while there was a slight decrease in the number of glucagon-positive cells in the islets (Figure 4o). In addition to the direct antidiabetic effect, the injection of the islets of Langerhans seeded on a DP scaffold may have a positive effect on the recovery processes of the recipient’s pool of actively functioning β-cells.

The glycemic parameters of the control group comprise the blood glucose levels of five rats during up to 14 days, and then, due to the death of one animal, the blood glucose levels of four rats in the course of up to 49 days, followed by data from three rats due to the death of another animal. The glycemic parameters of experimental group 1 consist of the blood glucose levels of five rats during up to 42 days, and then due to the death of one animal, the readings from four rats were taken into account. The glycemic parameters of experimental group 2 comprise the blood glucose levels of five rats from up to 28 days, and then, due to the death of one animal, the readings from four rats were taken into account.

A comparative analysis of changes in blood glucose levels in the control and experimental groups’ animals (Figure 5) showed that in experimental group 1, there was a noticeable decrease in glycemia levels after the islets of Langerhans injection, from 27.4 ± 1.8 mmol/L to 13.3 ± 1.0 mmol/L. Such indicators persisted for 7 weeks, after which there was an increase in glycemia to levels close to baseline values (before the islets of Langerhans injection). In experimental group 2, a more pronounced decrease in glycemia was noted versus experimental group 1 from 25.5 ± 1.9 mmol/L to 7.3 ± 0.9 mmol/L; the concentration of glucose in the blood of animals did not rise above 10.9 ± 2.3 mmol/L throughout the entire observation period. By week 10 of the experiment, the glucose level was 2.3 times lower than the baseline on average. In the experimental group 2 rats, after an injection of the islets of Langerhans seeded on a DP scaffold, a maximum decrease in the glycemia level with regard to the initial hyperglycemic parameters was observed by 71.4%; in the experimental group 1 rats, after an injection of islets without a scaffold, by 51.2%.

## 4. Discussion

One important strategy to help the islets cope with transplantation conditions is the development of biomaterial-based scaffolds to improve the microenvironment and enhance the viability/function of islets after transplantation. The restoration of the ECM microenvironment appears to reduce the physiological stress experienced by islets during and after isolation and consequently reduces islet cell death. It should be noted that most of the scaffold materials studied so far were based on synthetic polymers or hydrogels, which include poly(lactide-co-glycolide), polyethyleneglycol, polycaprolactone, polyglycolic acid, collagen gels, fibrin gels, alginate gels, etc. [13,14,15,16,17,18,19,22,41]. However, synthetic scaffolds may require additional modification with ECM components to provide a positive effect on islet survival and function.

Hydrogels obtained from natural materials (collagens, fibronectin, laminin, etc.) have better biocompatibility, consist of ECM components, and can be administered in an injectable form. Sackett et al. demonstrated the feasibility of creating a hydrogel for tissue-engineering pancreas from decellularized pancreatic tissue [42]. However, the technology for producing hydrogels requires a greater number of stages (steps), during which the tissue is subjected to harsh treatment with reagents (enzymes, acids), which ultimately affects the quality and quantity of the ECM components.

Previously, we conducted a comparative analysis of the effect of microheterogeneous collagen-containing hydrogel and a tissue-specific scaffold from a decellularized pancreas on the viability and function of rat islets of Langerhans in vitro. The advantage of using tissue-specific scaffold was revealed [33].

Scaffolds from completely decellularized organs, such as pancreas, lungs, liver, and spleen, are closest to the native ECM of pancreatic tissue [29,41]. An optimal scaffold obtained from decellularized pancreatic tissue should (1) meet the criteria for effective decellularization, (2) maximally preserve the native structure, (3) provide sites necessary for cellular adhesion and proliferation, (4) be uniformly populated with insulin-producing cells [28,30,41]. The presence of native proteins (various types of collagen, elastin, fibronectin and laminin) and cell adhesion factors in the decellularized pancreas scaffold allows for the creation of conditions for the prolonged vital activity of islet cells, thereby maintaining the islets critical mass necessary for implantation to patients with T1DM [43].

It should be noted that when assessing the composition of a DP scaffold, the preservation of GAGs was detected. GAGs are complex anionic unbranched heteropolysaccharides that represent the main structural and functional components of the connective tissue ECM and are involved in the modulation of various biochemical processes [44]. These molecules are involved in the regulation of vascular permeability, lipid metabolism, hemostasis and thrombosis, and interact with cells, growth factors and cytokines to modulate cell adhesion, migration, proliferation, and differentiation [27,45]. A number of decellularization protocols have shown the loss of GAGs as a result of harsh tissue processing [46,47,48]. The preservation of GAGs demonstrates the ability of the decellularization protocol to effectively remove DNA while carefully preserving the ECM composition.

Several analyses confirmed that our protocol effectively removes cellular material while preserving ECM proteins. DNA quantification demonstrated an obvious reduction in DNA compared with that of the natural tissue (14,058 [13,970; 14,786] ng/mg of tissue to 33 [26; 45] ng/mg of tissue, *p* <  0.05). It was found that the DP scaffold contained 0.92 [0.84; 1.16] µg/mg of sulfated GAGs and 273.7 [241.2; 303.0] µg/mg of collagen, while in the native pancreas, their number was 0.46 [0.44; 0.48] µg/mg and 3.1 [3.0; 4.0] µg/mg, correspondingly (Figure 2g,i). This fact was explained by an increase in the proportion of GAGs per unit mass of dried tissue due to the removal of cells in parenchymal organs, which was consistent with previously published data [49,50].

Our previous study [30] demonstrated the ability of the developed DP scaffold to support the adhesion and proliferation of human adipose-derived mesenchymal stromal cells (hADSCs). It also showed that the obtained DP scaffold in vitro facilitates both the islets’ viability and the maintenance of their secretory function for 7 days at a higher level versus the islets alone.

In this study, we investigated the effect of a microdispersed tissue-specific scaffold derived from human pancreatic tissue on the prolongation of islet function when injected intraperitoneally to rats with streptozotocin-induced T1DM.

In vivo experimental models are used in the studies of the pathophysiology of T1DM from onset to disease progression and also provide valuable information on the mechanism of various agents when studying the effectiveness of antidiabetic therapy. Most often, rodents—mice and rats—are used to model T1DM due to their availability, low cost of breeding, high reproduction rate, ethical acceptability, standardized sanitary environment, genetic similarity with human genes of more than 85%, as well as the effectiveness of genetic modification methods [51].

In animals, T1DM is often induced using chemicals (such as alloxan and STZ), which have diabetogenic properties that lead to the destruction of the islets of Langerhans insulin-producing β-cells, but preserve the function of the exocrine pancreas. STZ is a natural antibiotic that has specific toxicity and causes β-cell necrosis in laboratory animals [32,52]. There are two ways of injecting STZ to obtain a T1DM experimental model: a single injection of a high dose of the drug (60 mg/kg and above) [53] or multiple injections (mostly 2–7) of small doses of STZ (12–15 mg/kg/day) [54] with a time interval that can be adjusted in accordance with specific experimental tasks. The method of a STZ single injection has a high modeling speed and facilitates the short-term and easy formation of the T1DM model but, at the same time, leads to high mortality among experimental animals.

When modeling type I diabetes, the possibility of the diabetic status reversal in animals should be taken into account. It is necessary to carefully select diabetogenic agents and correct doses to reduce the self-healing incidence. Thus, it has been shown that STZ is preferable as a diabetogenic agent. When using alloxan, a temporary reversal of diabetic status was observed, which may lead to the misinterpretation of experimental results [55].

In our study, we chose a model of fractional injection of STZ (15 mg/kg/day) for 5 days, which allowed us to obtain a stable model of T1DM and exclude animal mortality during modeling, as well as avoid reversion of the diabetic status of animals.

Despite the many published protocols for obtaining scaffolds from decellularized tissues, a few research publications are known on the implantation of a tissue-engineered pancreas in animals with T1DM, formed on the basis of such scaffolds.

Wu et al. assessed in vivo the functions of β-cell-recellularized insulinoma MIN-6 bioscaffold from decellularized mouse pancreas and demonstrated the potential to reduce blood glycemia in experimental animals with T1DM [31].

Wang et al. showed in a model of mice with T1DM that constructs based on islets of Langerhans and a scaffold made from decellularized porcine pericardium allowed for the rapid reversal of the hyperglycemic condition in the host. The effect was achieved with a small number of islets: 250 islets/mouse [56].

Yu et al. demonstrated the fundamental possibility of a circulation-connected transplantation of a recellularized pancreas based on a scaffold produced from the tail of a decellularized rat pancreas. INSL-1E cells were used for recellularization [57].

Chandrika et al. decellularized a whole mouse pancreas, then recellularized it with mesenchymal stromal cells from human placenta and implanted it into mice with T1DM. The process of functional recovery of endocrine system took about 20 days when the mice started showing blood glucose reduction, though none achieved gluconormalization [58].

Citro et al. developed functional islet organs based on acellular lung matrixes, endothelial cells, and islets of Langerhans. The construct was formed in a perfusion bioreactor. It was shown that the implantation of the constructs into diabetic mice allows for the achievement of normoglycemia significantly faster and more effectively compared to islets that were transplanted without the use of devices [59].

It is worth noting that the recellularization of scaffolds with cells other than islets of Langerhans may be the first step in investigating the potential use of scaffolds to form tissue-engineered pancreas. Due to their size and multicellular complex composition, the uniform recellularization of large scaffolds derived from whole organs remains an unsolved problem. In addition, the transplantation of such a structure is a serious surgical intervention.

In our study, an alternative approach was considered, which consisted of combining the islets of Langerhans with a scaffold obtained from pancreas fragments. Such a finely dispersed scaffold with the preserved components of pancreatic tissue loaded with islets can be used to create an injectable form of a tissue-engineered pancreas, which will ensure a minimally invasive implantation and also the uniform population of the entire volume of the scaffold with islets, and promote the free diffusion of gasses and nutrients to the islet cells. In the future, to assess the potential therapeutic effectiveness of the introduction of islets with the developed scaffold, it is of interest to conduct a glucose load test to determine the degree of compensation for glucose metabolism impaired in diabetes.

## 5. Conclusions

For reliably interpreted results, it is necessary to select a relatively easily reproducible T1DM model with low mortality of experimental animals and a highly selective diabetogenic reagent to obtain stable hyperglycemic indices in rat blood (from 20 mmol/L to 33 mmol/L).

It was shown that the developed protocol for the decellularization of human pancreas fragments allows us to obtain a scaffold with a low DNA content (33 [26; 38] ng/mg of tissue, *p* <  0.05) and the preservation of GAG (0.92 [0.84; 1.16] µg/mg, *p* <  0.05) and fibrillar collagen (273.7 [241.2; 303.0] µg/mg, *p* <  0.05). The absence of a cytotoxic effect of the scaffold was revealed.

Using the streptozotocin-induced diabetic rat model, it was found that a tissue-specific fine-dispersed scaffold from decellularized pancreatic tissue provides pancreatic islets with a longer survival and effective function in vivo, which leads to a more pronounced antidiabetic effect compared to the injection of islets alone. The maximum decrease in glycemia levels relative to baseline values after the introduction of islets seeded on the DP scaffold was 71.4% and after the introduction of islets alone—51.2%.

The results obtained on the experimental tissue-engineered design of a pancreas allow us to hope for the possibility of creating a bio-artificial human pancreas based on a tissue-specific scaffold from decellularized human pancreatic tissue and the islets of Langerhans from postmortem donors, to partially or completely replace the lost pancreas endocrine function in patients with severe T1DM.

## Data Availability

The original contributions presented in the study are included in the article; further inquiries can be directed to the corresponding author.
